# 亚厘米肺腺癌临床特征及预后分析

**DOI:** 10.3779/j.issn.1009-3419.2019.08.04

**Published:** 2019-08-20

**Authors:** 嘉辉 宓, 少东 王, 晓 李, 冠潮 姜

**Affiliations:** 100044 北京，北京大学人民医院胸外科 Department of Thoracic Surgery, Peking University People's Hospital, Beijing 100044, China

**Keywords:** 亚厘米肺腺癌, 临床特点, 肺浸润性腺癌, 危险因素, 预后, Sub-centimeter lung adenocarcinoma, Clinical characteristics, Invasive lung adenocarcinoma, Risk factors, Prognosis

## Abstract

**背景与目的:**

随着肺癌筛查的逐渐推广，越来越多的患者被确诊为亚厘米(直径≤1 cm)肺腺癌。亚厘米肺腺癌多为早期肺癌，但目前关于亚厘米肺腺癌的研究仍不充分。本研究针对亚厘米肺腺癌患者临床特征及预后进行分析，为该类患者的诊疗提供依据。

**方法:**

回顾性分析2012年1月-2016年12月北京大学人民医院经胸腔镜手术病理确诊为亚厘米肺腺癌患者的临床及预后资料。根据结节影像学特征将患者分为纯磨玻璃结节(pure ground-glass nodules, pGGN)、混杂性磨玻璃结节(mixed ground-glass nodules, mGGN)和实性结节(solid nodules, SN)组，对比三组患者临床特征并对不同直径结节行亚组分析。此外，通过多因素分析筛选亚厘米肺浸润性腺癌的独立危险因素。

**结果:**

本组共182例患者，中位年龄54(27-75)岁。男性57例，女性125例。女性亚厘米肺腺癌患者无吸烟史比例显著高于男性(*P* < 0.001)。所有1 mm-10 mm pGGN、1 mm-5 mm mGGN及1 mm-5 mm SN患者术后病理除原发灶外无其他阳性发现。46例6 mm-10 mm mGGN患者中有3例侵犯胸膜，1例发现脉管癌栓。39例6 mm-10 mm SN患者中有5例侵犯胸膜，2例发现脉管癌栓，2例出现淋巴结转移。侵犯胸膜、发现脉管癌栓或淋巴结转移的患者其病理类型均为浸润性腺癌。多因素*Logistic*回归分析发现吸烟史、既往肿瘤病史、mGGN、SN和肿瘤直径 > 5 mm是病理为肺浸润性腺癌的独立危险因素。中位随访时间44(22-82)个月，全组患者5年无复发生存率100.0%，总生存率98.9%。

**结论:**

亚厘米肺腺癌患者发病年龄相对较小。影像学表现为6 mm-10 mm mGGN和6 mm-10 mm SN的亚厘米肺浸润性腺癌患者存在侵犯胸膜或淋巴结转移可能。吸烟史、既往肿瘤病史、mGGN、SN和肿瘤直径 > 5 mm为亚厘米肺浸润性腺癌的独立危险因素。对于亚厘米肺腺癌患者，早期发现并采取合适且有效的外科干预可获得良好的预后。

## Abstract (en)

**Background and objective:**

With the increase of lung cancer screening, more and more patients have been diagnosed as sub-centimeter (≤1 cm) lung adenocarcinoma. Sub-centimeter lung adenocarcinoma is mostly early stage lung cancer, but the research on sub-centimeter lung adenocarcinoma is still insufficient. This study analyzed the clinical characteristics and prognosis of patients with sub-centimeter lung adenocarcinoma in order to provide the basis for the diagnosis and treatment of such patients.

**Methods:**

A retrospective study was performed to analyze patients with sub-centimeter lung adenocarcinoma who underwent VATS in Peking University People's Hospital from January 2012 to December 2016. Patients were divided into pure ground-glass nodules (pGGN) group, mixed ground-glass nodules (mGGN) group and solid nodules (SN) group according to the features of nodular imaging. The clinical characteristics of the three groups were compared and the subgroup analysis of nodules in different diameter was performed. We also performed multivariate logistic regression analyses to identify the risk factors for sub-centimeter lung invasive adenocarcinoma.

**Results:**

The study included 182 patients (57 men and 125 women) with a median age of 54 (27-75) years. Female sub-centimeter lung adenocarcinoma patients had a significantly lower proportion of non-smoking history than males (*P* < 0.001). All patients with 1 mm-10 mm pGGN, 1 mm-5 mm mGGN and 1 mm-5 mm SN had no other pathologically positive findings except for the primary lesion. Of the 46 patients with 6 mm-10 mm mGGN, 3 had pleural invasion and 1 had vascular tumor thrombus. Of the 39 patients with 6 mm-10 mm SN, 5 had pleural invasion, 2 had vascular tumor thrombus and 2 had lymph node metastasis. The pathological type in each patient with pleural invasion, vascular tumor thrombus or lymph node metastasis was invasive adenocarcinoma. *Logistic* regression analysis indicated that smoking history (OR=4.727, *P*=0.009), previous tumor history (OR=3.408, *P*=0.015), mGGN (OR=3.735, *P*=0.004), SN (OR=8.921, *P* < 0.001) and tumor diameter > 5 mm (OR=4.241, *P*=0.001) were independent risk factors for sub-centimeter lung invasive adenocarcinoma. The median follow-up time was 44 (22-82) months. The 5-year recurrence-free survival rate was 100.0% and the overall survival rate was 98.9%.

**Conclusion:**

Patients with sub-centimeter lung adenocarcinoma have a relatively earlier onset age. Sub-centimeter lung invasive adenocarcinoma patients with 6 mm-10 mm mGGN and 6 mm-10 mm SN may be involved in pleural invasion or lymph node metastasis. Smoking history, previous tumor history, mGGN, SN and tumor diameter > 5 mm are independent risk factors for sub-centimeter lung invasive adenocarcinoma. For patients with sub-centimeter lung adenocarcinoma, early detection and appropriate surgical intervention can lead to a good prognosis.

随着肺癌筛查的逐渐推广，进行低剂量胸部计算机断层扫描(computed tomography, CT)筛查人数逐年上升，越来越多亚厘米(直径≤1 cm)肺腺癌患者被早期发现并得到及时的外科干预。亚厘米肺腺癌影像学表现不同，恶性程度不同，处理干预原则不同，预后也不尽相同。影像学表现为亚厘米纯磨玻璃结节(pure ground-glass nodules, pGGN)多为癌前病变，预后较好，但仍有部分亚厘米肺腺癌患者存在转移或复发的风险^[1, 2]^。然而，目前对于亚厘米肺腺癌的临床特点研究仍不充分，外科处理方法也尚无定论。因此，本研究回顾总结182例亚厘米肺腺癌患者临床特征及预后情况，为该类患者今后的诊疗提供依据。

## 1 资料和方法

### 1.1 一般资料

回顾性分析2012年1月-2016年12月北京大学人民医院收治的亚厘米肺腺癌患者。纳入标准：①肺内孤立性结节; ②接受胸腔镜手术治疗; ③术后病理证实为肺腺癌; ④具有完整临床资料，包括胸部CT、术后病理、随访资料等。排除标准：石蜡病理证实为肺良性结节或转移癌。共入组182例患者，男性57例(31.3%)，女性125例(68.7%)，中位年龄54(27-75)岁。22例患者既往存在吸烟史(吸烟总数超过100支，包括就诊前仍在吸烟以及已戒烟患者)。以咳嗽、咳痰等主诉就诊患者16例(8.8%)，以胸闷或胸痛主诉就诊患者5例(2.7%)，以发热为主诉就诊患者3例(1.6%)，无症状体检发现肺结节患者158例(86.8%)。

### 1.2 分组方法

根据肺部结节不同影像学特征进行分组：(1)纯磨玻璃结节：肺内存在呈圆形或类圆形的密度增高影，但病变密度不足以掩盖其中走行的血管和支气管影; (2)混杂性磨玻璃结节(mixed ground-glass nodules, mGGN)：肺内结节在纯磨玻璃影的基础上存在部分实性成分。对于该类患者，根据薄层CT结果进一步计算其实性成分最大直径与肿瘤最大直径比值(consolidation/tumor ratio, CTR); (3)实性结节(solid nodule, SN)：无磨玻璃影存在，完全为实性成分。此外，根据肿瘤直径分为1 mm-5 mm和6 mm-10 mm两组，行进一步亚组分析。

### 1.3 术前评估及手术方法

本中心亚厘米肺部结节患者接受手术治疗遵循以下原则：①随访过程中结节增大、出现实性成分或实性成分增多者; ②患者心理压力大，具有强烈手术意愿，继续观察随访严重影响生活质量者。手术方法：所有患者均在双腔气管插管全身麻醉下行电视胸腔镜手术。对于术中难以准确定位的小结节，根据我中心肺部小结节微弹簧圈定位标准进行术前定位，其余病灶直接行手术切除。对于周围型结节，先行楔形切除，切缘距离病灶边缘≥2 cm，术中送快速冰冻病理，根据冰冻病理结果决定下一步手术方式，尤其SN且冰冻病理明确回报为浸润性腺癌，通常继续行肺叶切除加淋巴结清扫术，如为pGGN且冰冻病理报告为贴壁生长的癌通常只进行楔形切除加淋巴结采样。对于位置较深的结节(中央型结节)按照R0切除并尽可能保留肺组织的原则行肺段切除或者肺叶切除，同样根据术中冰冻结果决定是否进一步行淋巴结清扫。

### 1.4 随访方法

收集所有入组患者临床数据及术后病理结果。病理分类参照2015版世界卫生组织(World Health Organization, WHO)肺癌组织学分类标准，肺癌分期参照第8版美国癌症联合委员会(American Joint Committee on Cancer, AJCC)肺癌分期标准。所有患者术后每6个月门诊随访，内容包括体格检查，胸部平扫CT，必要时加做其他相关检查。对于不能定期于我院门诊进行随访的患者，定期于当地进行检查并电话汇报至我中心随访专员。无复发生存期定义为手术结束直至出现局部复发或远处转移的时间。总生存期定义为手术结束到死亡的时间，或截止到最后一次随访的时间。

### 1.5 统计学处理

采用SPSS 24.0统计学软件处理。计量资料符合正态分布的数据采用独立样本*t*检验; 若不符合正态分布则采用*Mann-Whitney U*检验; 计数资料采用*χ*^2^检验或*Fisher*确切概率法检验; 采用*Logistic*回归模型进行多因素分析确定独立危险因素; *Kaplan-Meier*法进行生存分析。*P* < 0.05为差异有统计学意义。

## 2 结果

### 2.1 三组患者一般资料及手术情况

20例男性(35.1%, 20/57)和2例女性(1.6%, 2/125)患者既往有吸烟史，女性亚厘米肺腺癌患者无吸烟史比例显著高于男性(*P* < 0.001)。全部手术均在电视胸腔镜下进行，80例肺楔形切除，23例行肺段切除，79例肺叶切除，所有楔形切除手术切缘距病变2 cm以上。三组患者一般资料及手术情况见表 1。三组患者在年龄、性别、吸烟史、术前肺功能评估及肺叶分布上无显著差异。在手术方式处理上SN组患者行肺叶切除比例较其他两组患者明显更高(*P*=0.004)。

**1:** 肺亚厘米pGGN、mGGN以及SN患者一般资料及手术情况 Baseline and operation characteristics in patients with sub-centimeter pGGN, mGGN and SN

Variables	pGGN (*n*=61)	mGGN (*n*=68)	SN (*n*=53)	*P*
Age (yr)	52.9±9.8	53.8±10.4	56.0±10.0	0.246
Gender				0.648
Male	17 (27.9%)	24 (35.3%)	16 (30.2%)	
Female	44 (72.1%)	44 (64.7%)	37 (69.8%)	
Smoking history				0.195
Negative	55 (90.2%)	62 (91.2%)	43 (81.1%)	
Positive	6 (9.8%)	6 (8.8%)	10 (18.9%)	
Pulmonary function				
FEV_1_ (L)	2.76±0.72	2.64±0.68	2.61±0.66	0.497
FEV_1_%	91.83±13.90	89.18±13.21	87.35±13.10	0.293
DLCO SB%	81.51±14.44	81.41±16.00	78.96±15.12	0.654
Operation				0.004
Wedge resection	34 (55.7%)	34 (50.0%)	12 (22.6%)	
Segmentectomy	8 (13.1%)	7 (10.3%)	8 (15.1%)	
Lobectomy	19 (31.1%)	27 (39.7%)	33 (62.3%)	
Tumor location				0.860
RUL	16 (26.2%)	24 (35.3%)	19 (35.8%)	
RML	9 (14.8%)	8 (11.8%)	6 (11.3%)	
RLL	4 (6.6%)	7 (10.3%)	6 (11.3%)	
LUL	18 (29.5%)	18 (26.5%)	15 (28.3%)	
LLL	14 (23.0%)	11 (16.2%)	7 (13.2%)	
Values are Mean±SD or *n* (%). pGGN: pure ground-grass nodules; mGGN: mixed ground-grass nodules; SN: solid nodules; FEV_1_: forced expiratory volume in 1 second; FEV_1_%: forced expiratory volumein 1 second of predicted; DLCO SB%: diffusing capacity for carbon monoxide of predicted; RUL: right upper lobe; RML: right middle lobe; RLL: right lower lobe; LUL: left upper lobe; LLL: left lower lobe.				

### 2.2 三组患者不同直径结节病理及分期情况

三组患者1 mm-5 mm和6 mm-10 mm结节病理及分期对比见表 2。1 mm-5 mm pGGN患者术后病理均为原位腺癌或微浸润腺癌。6 mm-10 mm pGGN患者相对于1 mm-5 mm pGGN患者Ia1期比例更高(*P*=0.025)。1 mm-10 mm pGGN、1 mm-5 mm mGGN及1 mm-5 mm SN患者术后病理除原发灶外无其他阳性发现。46例6 mm-10 mm mGGN患者中有3例侵犯胸膜，1例发现脉管癌栓，侵犯胸膜或发现脉管癌栓mGGN患者CTR均大于0.5，且病理类型均为浸润性腺癌; 39例6 mm-10 mm SN患者中有5例侵犯胸膜，2例发现脉管癌栓，2例出现淋巴结转移，侵犯胸膜、发现脉管癌栓或淋巴结转移SN患者病理类型同样均为浸润性腺癌。

**2:** 三组患者1 mm-5 mm和6 mm-10 mm结节病理及分期对比 Pathological and staging comparison of 1 mm-5 mm and 6 mm-10 mm nodules in three groups

Variables	pGGN (*n*=61)				mGGN (*n*=68)				SN (*n*=53)		
	1 mm-5 mm (*n*=19)	6 mm-10 mm (*n*=42)	*P*		1 mm-5 mm (*n*=22)	6 mm-10 mm (*n*=46)	*P*		1 mm-5 mm (*n*=14)	6 mm-10 mm (*n*=39)	*P*
Pleural invasion			1.000				0.546				0.309
PL0	19 (100.0%)	42 (100.0%)			22 (100.0%)	43 (93.5%)			14 (100.0%)	34 (81.2%)	
PL1	0 (0.0%)	0 (0.0%)			0 (0.0%)	3 (6.5%)			0 (0.0%)	5 (12.8%)	
Tumor thrombus			1.000				1.000				0.604
Negative	19 (100.0%)	42 (100.0%)			22 (100.0%)	45 (97.8%)			14 (100.0%)	37 (94.9%)	
Positive	0 (0.0%)	0 (0.0%)			0 (0.0%)	1 (2.1%)			0 (0.0%)	2 (5.1%)	
Nodal involvement			1.000				1.000				0.604
N0	19 (100.0%)	42 (100.0%)			22 (100.0%)	46 (100.0%)			14 (100.0%)	37 (94.9%)	
N2	0 (0.0%)	0 (0.0%)			0 (0.0%)	0 (0.0%)			0 (0.0%)	2 (5.1%)	
Histology			0.021				0.106				0.124
AIS	12 (63.2%)	13 (31.0%)			6 (27.3%)	6 (13.0%)			2 (14.3%)	3 (7.7%)	
MIA	7 (36.8%)	20 (47.6%)			11 (50.0%)	18 (39.1%)			6 (42.9%)	8 (20.5%)	
IAC	0 (0.0%)	9 (21.4%)			5 (22.7%)	22 (47.8%)			6 (42.9%)	28 (71.8%)	
Stage			0.025				0.230				0.508
0	12 (63.2%)	13 (31.0%)			6 (27.2%)	6 (13.0%)			2 (14.3%)	3 (7.7%)	
Ia1	7 (36.8%)	29 (69.0%)			16 (72.7%)	37 (80.4%)			12 (85.7%)	29 (74.4%)	
Ib	0 (0.0%)	0 (0.0%)			0 (0.0%)	3 (6.5%)			0 (0.0%)	5 (12.8%)	
IIIa	0 (0.0%)	0 (0.0%)			0 (0.0%)	0 (0.0%)			0 (0.0%)	2 (5.1%)	
Values are *n* (%). AIS: adenocarcinoma *in situ*; MIA: minimally invasive adenocarcinoma; IAC: invasive adenocarcinoma.											

### 2.3 亚厘米肺浸润性腺癌危险因素分析

亚厘米肺浸润性腺癌与恶性程度相对较低的肺腺癌(原位腺癌和微浸润腺癌)临床特征对比结果见(表 3)。将年龄、性别、吸烟史、既往肿瘤病史、结节影像学特征和肿瘤直径进一步纳入*Logistic*回归模型进行多因素分析，结果显示吸烟史、既往肿瘤病史、mGGN、SN和肿瘤直径 > 5 mm为亚厘米肺浸润性腺癌的独立危险因素(表 4)。

**3:** 亚厘米肺浸润性腺癌和恶性程度相对较低的肺腺癌(原位腺癌和微浸润腺癌)临床特征对比 Comparison of clinical characteristics between sub-centimeter lung IAC and less malignant adenocarcinoma (AIS and MIA)

Variables	AIS and MIA (*n*=112)	IAC (*n*=70)	*P*
Age (yr)	55.8±10.5	53.1±9.8	0.076
Gender			0.095
Male	30 (26.8%)	27 (38.6%)	
Female	82 (73.2%)	43 (61.4%)	
Smoking history			0.002
Negative	105 (93.8%)	55 (78.6%)	
Positive	7 (6.2%)	15 (21.4%)	
Family history of lung cancer			0.399
Negative	102 (91.1%)	61 (87.1%)	
Positive	10 (8.9%)	9 (12.9%)	
Clinical symptoms			0.729
Negative	98 (87.5%)	60 (85.7%)	
Positive	14 (12.5%)	10 (14.3%)	
Preoperativecomplications			0.580
Negative	78 (69.6%)	46 (65.7%)	
Positive	34 (30.4%)	24 (34.3%)	
Previous tumor history			0.005
Negative	103 (92.0%)	54 (77.1%)	
Positive	9 (8.0%)	16 (22.9%)	
Tumor characteristics			< 0.001
pGGN	51 (45.5%)	10 (14.3%)	
mGGN	41 (36.6%)	27 (38.6%)	
SN	20 (17.9%)	33 (47.1%)	
Tumor diameter			0.001
≤5 mm	44 (39.3%)	11 (15.7%)	
> 5 mm	68 (60.7%)	59 (84.3%)	
Tumor location			0.761
RUL	35 (31.2%)	24 (34.3%)	
RML	14 (12.5%)	9 (12.9%)	
RLL	10 (9.0%)	7 (10.0%)	
LUL	30 (26.8%)	21 (30.0%)	
LLL	23 (20.5%)	9 (12.9%)	
Values are Mean±SD or *n* (%).			

**4:** 亚厘米肺浸润性腺癌多因素分析 Multivariate analysis for risk factors associated with sub-centimeter lung invasive adenocarcinoma

Variables	*β*	OR	95%CI	*P*
Smoking history	1.553	4.727	1.484-10.051	0.009
Previous tumor history	1.226	3.408	1.271-9.138	0.015
mGGN	1.318	3.735	1.528-9.130	0.004
SN	2.188	8.921	3.432-23.189	< 0.001
Tumor diameter > 5 mm	1.445	4.241	1.837-9.788	0.001
mGGN: mixed ground-grass nodules; SN: solid nodules.				

### 2.4 术后治疗及预后情况

排除1例肺叶切除围手术期因肺栓塞死亡患者，中位随访时间为44(22-82)个月。0期及Ia1期患者术后持续复查无其他治疗; 8例Ib期患者有2例术后接受含铂双药方案辅助化疗4周期，其余6例术后无其他治疗; 2例IIIa期患者术后接受含铂双药方案辅助化疗4周期并接受术后放疗。随访期间有1例行肺叶切除Ia1期患者于术后16个月死于重症肺炎。全组患者5年无复发生存率100.0%，总生存率98.9%。

## 3 讨论

张思维等^[3]^统计2014年中国肿瘤登记地区肺癌发病中位年龄在65岁-69岁之间，而本研究中亚厘米肺腺癌患者发病中位年龄仅为54岁，低于整体肺癌人群。此外，本研究发现女性亚厘米肺腺癌非吸烟患者比例显著高于男性，究其原因一方面可能是由于雌激素-雌激素受体介导的信号通路促进了女性肺腺癌的发生^[4]^，另一方面部分中国非吸烟女性长期被动吸烟史(香烟烟雾、烹饪烟雾)与肺腺癌的发生也可能具有相关性^[5]^。因此，对于发现肺部亚厘米结节但既往无主动吸烟史的中老年女性患者，医师在临床中应仔细询问其被动吸烟史并提高肺癌的风险意识。

亚肺叶切除治疗早期肺腺癌已经被越来越多外科医生所接受，有大量研究^[6-8]^指出亚肺叶与肺叶切除在早期肺腺癌治疗中无显著差异。对于亚厘米肺腺癌，肿瘤位置是决定手术方式的重要因素，且实施亚肺叶切除的前提是要求获得足够的手术切缘^[9]^。亚厘米pGGN肺腺癌患者组织病理学类型多为原位腺癌或微浸润腺癌等早期病变，生长缓慢且通常无胸膜侵犯、脉管癌栓以及淋巴结转移发生，预后较好^[2, 10]^，本研究61例亚厘米pGGN肺腺癌患者统计结果与先前研究一致。此外，肿瘤直径与肺癌侵袭性同样存在相关性，本组1 mm-5 mm mGGN和1 mm-5 mm SN肺腺癌患者未观察到除原发灶以外任何阳性病理结果。因此，对于1 mm-10 mm pGGN、1 mm-5 mm mGGN及1 mm-5 mm SN患者，在经过术前评估决定手术后，可积极采取亚肺叶切除的手术方式，在保证手术效果的前提下减少患者肺损失，提高术后生存质量。

本组患者中，有2例直径6 mm-10 mm SN患者出现了淋巴结转移。其中1例患者术中首先行楔形切除及淋巴结采样，术中冰冻病理提示浸润性腺癌，9组淋巴结可见癌转移。遂改行肺叶切除加淋巴结清扫。术后石蜡病理提示为浸润性中分化腺癌，其中4组、7组、9组、12组淋巴结可见癌转移。另一患者因肿瘤位置较深，术中直接行肺叶切除，术中冰冻病理提示浸润性腺癌，遂行淋巴结清扫，术后病理为浸润性中-低分化腺癌，4组、7组、8组、12组淋巴结可见癌转移。既往有研究^[11]^表明影像学表现为SN的肺癌其恶性程度相对更高，Sakurai等^[1]^也发现亚厘米结节中只有SN才会发生淋巴结转移，但遗憾的是该研究未进一步对发生淋巴结转移的亚厘米SN的直径行亚组分析。除此之外，本研究6 mm-10 mm mGGN肺腺癌患者虽然无淋巴结转移发生，但CTR > 0.5的患者中仍有3例侵犯胸膜，1例发现脉管癌。mGGN中的实性成分常被认为是肿瘤浸润性生长的部分，随着CTR的增大，发生淋巴结转移或局部复发的概率也随之增高^[12, 13]^。因此，本研究认为对于CTR > 0.5的6 mm-10 mm mGGN和6 mm-10 mm SN患者，可采取更为积极的外科处理。

肺浸润性腺癌相对于肺原位腺癌或微浸润腺癌其生物学特征更具侵袭性^[14]^。因此，本研究采用多因素分析的方式研究肺浸润腺癌的危险因素。结果显示吸烟史、既往肿瘤病史、mGGN、SN和肿瘤直径 > 5 mm为亚厘米肺浸润性腺癌的独立危险因素，与既往研究结果基本相符^[15]^。此外，Kinsey等^[16]^的研究发现结节位置也是肺恶性肿瘤的独立危险因素——浸润性腺癌更倾向发生于肺上部区域。但本研究未发现亚厘米肺浸润性腺癌的发生与结节位置存在明显相关性。因此，本研究不支持将亚厘米肺结节的发生位置作为肺浸润性腺癌的预测因素。

本组患者中有1例72岁女性患者在接受肺叶切除5 d后因急性肺栓塞死亡。Li等^[17]^对11, 474例肺癌患者进行多因素分析发现，超过66岁并接受外科干预是发生术后肺栓塞的独立危险因素。此外，肥胖、长期卧床、手术创伤、恶性肿瘤等均为发生急性肺栓塞的高危因素。虽然急性肺栓塞早期往往伴随疼痛、呼吸困难、肺部干湿性啰音、心率加快等临床症状，但在亚厘米肺腺癌手术术后，这些非特异性临床表现往往容易被忽略。因此，对于具有急性肺栓塞高危因素的亚厘米肺腺癌患者，外科医师仍应提高肺栓塞风险意识，在对高危患者早期干预的同时密切关注其病情变化。

本组患者中，接受手术治疗的亚厘米肺腺癌患者其5年无复发生存率及总生存率分别达到100.0%和98.9%。其中0期及Ia1期患者术后可基本达到根治目的。对于Ib期患者术后是否应行辅助化疗目前存在争议，有研究^[18]^表明，对于直径≤2 cm的肺腺癌患者，肿瘤是否侵犯胸膜与肿瘤复发以及总生存期长短无明显相关性。本研究中8例侵犯胸膜的Ib期亚厘米肺腺癌患者仅有2例术后接受了含铂双药辅助化疗方案，其余6例患者术后未接受任何治疗，8例Ib期患者术后均无转移或复发。该结果提示侵犯胸膜的Ib期亚厘米肺腺癌患者彻底切除病灶后不接受术后辅助化疗也能获得较好预后。此外，2例IIIa期患者术后接受含铂双药方案辅助化疗4周期和辅助放疗，截至最后一次随访2例IIIa期患者无复发生存期已分别达到52个月和71个月，获得了良好的预后。

本研究还存在着一些不足：首先，回顾性研究存在选择偏倚; 其次，手术方式的选择在一定程度上与医师经验或患者意愿相关。在今后研究中可开展更大规模的前瞻性、多中心临床研究以克服上述不足。

本研究表明：亚厘米肺腺癌患者流行病学特征与整体肺癌人群存在差异，发病年龄相对较小。虽然大多数亚厘米肺腺癌均为早期肺癌，但6 mm-10 mm mGGN和6 mm-10 mm SN亚厘米肺浸润性腺癌仍存在侵犯胸膜或淋巴结转移可能。此外，吸烟史、既往肿瘤病史、mGGN、SN和肿瘤直径 > 5 mm为亚厘米肺浸润性腺癌的独立危险因素，需要在今后的临床实践中加以重视。从预后情况来看，亚厘米肺腺癌患者行外科干预总体效果良好，早期发现并采取合适且有效的外科干预可获得良好的预后。
